# Supplementary material for: Genome-Wide Identification of Maize Protein Arginine Methyltransferase Genes and Functional Analysis of ZmPRMT1 Reveal Essential Roles in Arabidopsis Flowering Regulation and Abiotic Stress Tolerance
Source: Int J Mol Sci. 2022 Oct 24;23(21):12793. doi: 10.3390/ijms232112793 (PMC9655960; doi:10.3390/ijms232112793)
Supplement: Supplementary file 1 [file ijms-23-12793-s001.zip › ijms-1966559-supplementary.pdf]

Supplementary materials

Figure S1 PCR electrophoresis of *ZmPRMT1* genes.

(A) *ZmPRMT1* gene clone electrophoresis diagram. M1:250-I Marker, A1-2: ***ZmPRMT1*** **gene.**

(B) Recombinant plasmid bienzyme-cut electrophoresis diagram. M2:250-III Marker, B1-2: ***ZmPRMT1*** **gene.**

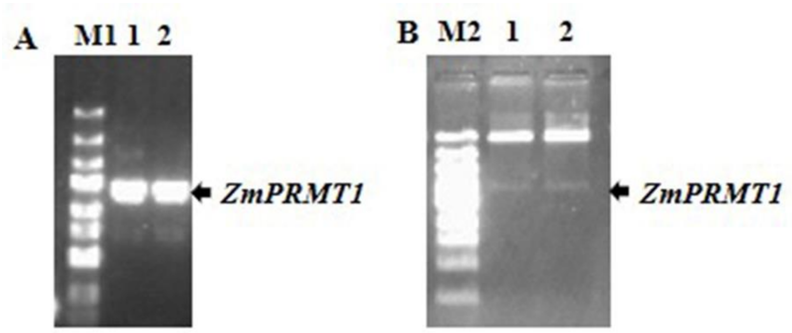

Figure S2 PCR electrophoresis of Hygromycin gene of 16 over-expression lines.

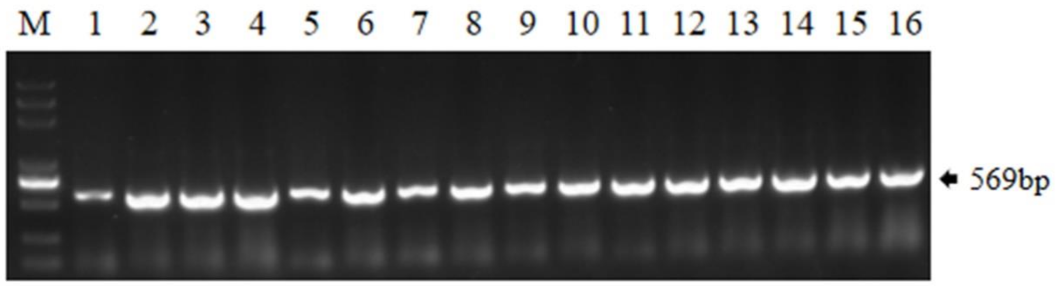

Figure S3 Alignment of the amino acid sequences of ZmPRMT1 protein and AtPRMT5 protein.

```
ZmPRMT1    .................MILTLQAAEETGRKLKVYAVEKN    23
AtPRMT5    SELTTVLMVVGAGRGPLVRASLQAAEETDRKLKVYAVEKN   400
Consensus                       lqaaeet rklkvyavekn

ZmPRMT1    PNAVITLHSLIKLEGWESLVTVISSDMRCWDAPEKADILV    63
AtPRMT5    PNAVVTLHNIVKMEGWEDVVTIISCDMRFWNAPEQADILV   440
Consensus  pnav tlh l k egwe  vt is dmr w ape adilv

ZmPRMT1    SELLGSFGDNELSPECLDGAQRFLKPDGISIPSSYTSFIQ   103
AtPRMT5    SELLGSFGDNELSPECLDGAQRFLKPDGISIPSSYTSFIQ   480
Consensus  sellgsfgdnelspecldgaqrflkpdgisipssytsfiq

ZmPRMT1    PITASKLHDDIKAHKDIAHFETAYVVKLHRIATLAPPQQV   143
AtPRMT5    PITASKLYNDVKAHKDLAHFETAYVVKLHSVAKLAPSQSV   520
Consensus  pitaskl  d kahkd ahfetayvvklh  a lap q v

ZmPRMT1    FTFTHPNFSPNASNQRYTKLQFEMLPDMGSCLVHGFAGYF   183
AtPRMT5    FTFTHPNFSTKVNNQRYKKLQFSLPSDAGSALVHGFAGYF   560
Consensus  ftfthpnfs    nqry klqf    d gs lvhgfagyf

ZmPRMT1    DSVLYKDVHLGIEPNTATPNMFSWFPIFFPLRKPIYVPEG   223
AtPRMT5    DSVLYKDVHLGIEPTTATPNMFSWFPIFFPLRKPVEVHPD   600
Consensus  dsvlykdvhlgiep tatpnmfswfpiffplrkp  v

ZmPRMT1    SPIEVHFWRCCAPTKVWYEWAVTTPTPSPIHNSNGRSYWV   263
AtPRMT5    TPLEVHFWRCCGSSKVWYEWSVSSPTPSPMHNTNGRSYWV   640
Consensus   p evhfwrcc   kvwyew v  ptpsp hn ngrsywv

ZmPRMT1    G                                          264
AtPRMT5    G                                          641
Consensus  g
```

Figure S4 Alignment of the amino acid sequences of PRMT proteins in maize.

```
ZmPRMT1    ................................................................................   0
ZmPRMT2    ................................................................................   0
ZmPRMT3    MASSPDLFPNVAFSDVSAAAP...AAEATAAFGLGATTGAPRLCLVKSGKDEAEPTVDIDLADAQVFKLGPMEWLCVCDE  77
ZmPRMT4    MASSPDLFPNVTFSDVSEAAAGPAAEGATAAFGLGAATGAPRLSLVKA...EAEPTVEIDLADAQVFKLGPTEWLCVCDE  77
ZmPRMT5    ................................................................................   0
ZmPRMT6    ................................................................................   0
ZmPRMT7    ................................................................................   0
ZmPRMT8    ................................................................................   0
Consensus

ZmPRMT1    ................................................................................   0
ZmPRMT2    ................................................................................   0
ZmPRMT3    SEAKAGVEE...............KSFSRAIKVVLRTEAESKAFSLAFQQWKHQVISGKAGERLENGSIIASKSKFDTKI 142
ZmPRMT4    SEAKDGVEEYVLNGYMLTLSSLRQKSFSRAIKVVLRTEAESKAFSLAFQRWQHQVISGKAGERLENGSIIASKNKFDTKI 157
ZmPRMT5    ................................MLPSHLNGHSALGR.RRPRLS..........AAAGESPAAAGVEAAAP  37
ZmPRMT6    ................................................................................   0
ZmPRMT7    ................................MFTGAAGGIGNLPRPRRPRLGGIGHGGVMVSPQGQVALGAHPHLAAPP  48
ZmPRMT8    ................................................................................   0
Consensus

ZmPRMT1    ................................................................................   0
ZmPRMT2    ..........................................................MLMAKCRTLQEENEEIGTMASE  22
ZmPRMT3    EASSAKMYFHYYGQLLHQQNMLQDFVRTGTYYAAVMENRSDFEGRVVVDVGAGSGILSLFAAQAGARHVYAVEASEMAEN 222
ZmPRMT4    EASSAKMYFHYYGQLLHQQNMLQDFVRTGTYYAAVMENRSDFEGRVVVDVGAGSGILSLFAVQAGARHVYAVEASEMAEH 237
ZmPRMT5    LEEHDRIYFQAYSHLGIHETMIKDRVRTDAYHAAIMRHQKFIEGKVVLDVGCGTGILSVFCARAGAKRVYAVEACEIVVQ 117
ZmPRMT6    ........................................................MSLLCS.....QVYAVDASDIAFQ  19
ZmPRMT7    CTDYDVAYFKAYSHIGVHEEMLKDHVRTSTYRNAIMHHKDLISGKVVLDVGCGTGVLSIFCAFAGATRVYAVDASDIAFQ 128
ZmPRMT8    ....................MLKDVVRTKTYQNVITQSSFLIKNKVVLDVGAGTGILSLFCAKAGAKHVYAIECSQMADM  60
Consensus

ZmPRMT1    .....................MRCWDA.PEKADILVSELLGSFGDNE.LSPECLDGAQRFLKPDGISIPSSYTSFIQPIT  57
ZmPRMT2    G.KIHELGMKIAVLKSQNNELRNQFDVLYKHMDGVTNDVERSNELVS.ILQEELEAKDLELTRLKEVLAQKGATQDAPVE 100
ZmPRMT3    AQRLMSGNPSLGQRITVIRGKVEEVEL.PEKADILISEPMGTLLVNERMLESYVIARDRFLAPDGKMFPTTGRIHMAPFS 301
ZmPRMT4    AQRLISGNPSLGHRITVIKGKVEEVVL.PEKADILISEPMGTLLVNERMLESYVIARDRLLAPDGKMFPTTGRIHMAPFS 316
ZmPRMT5    AREIVKAN.NLTDQIVVIHGRVEDVDL.EEKVDVIISEWMGYMLLYESMLPSVLFARDKWLKPGGLILPSHATLFMAPVT 195
ZmPRMT6    AMEIVREN.ELSDKVVVLHGRIEDVDI.EEKVDVIISEWMGYMLLYESMLGSVIFARDKWLKPGGLILPSHASLYMAPVT  97
ZmPRMT7    AMEIVREN.ELSDKVVVLHGRIEDVDI.EEKVDVIISEWMGYMLLYESMLGSVIFARDKWLKPGGLILPSHASLYMAPVT 206
ZmPRMT8    AQEIVKSN.GYSDVITVIKGKVEEIELPVPKVDVIISEWMGYFLLFENMLNTVLYARDKWLADGGVVLPDRTSLRLTAIE 139
Consensus                                  d                           l

ZmPRMT1    ASKLHDDIK......AHKDIAHFETAYVVKLHRIATLAPP.............QQVRDLICCVCFIGSDQIYLCHEIWLS 118
ZmPRMT2    TSDDAGNGQ.....EADSDTLQVKAES..................................................... 122
ZmPRMT3    DEYLYVEIANKALFWQQHNFFGVDLTPLHGSAFQGYFSQPVVDAFDPR.LLISPPIYHTLDFTSMKEEELYEIDIPLSFV 380
ZmPRMT4    DEYLYVEMANKALFWQQHNFFGVDLTPLHGSAFQGYFSQPVVDAFDPR.LLISPPTYHTLDFTSMKEEELYEINIPLSFV 395
ZmPRMT5    NRERYEDSV.....DFWRDVYGINMSALVPLAKKFTSEEPSIETIGGENVISWPSVFKRFDCYNFTAEEFKSITSKYKVS 270
ZmPRMT6    NSQRYHDSI.....YFWRDVYGIKMSSMMPLAKLCAFMEPSVETISGENVLTWPAVVAQVDCYTIQAQKLETITAAFKFT 172
ZmPRMT7    NSQRYHDSI.....YFWRDVYGIKMSSMMPLAKLCAFMEPSVETISGENVLTWPAVVAQVDCYTIQAQKLETITAAFKFT 281
ZmPRMT8    DAEYK........................................................................... 144
Consensus

ZmPRMT1    PLLV............................................................................ 122
ZmPRMT2    ................................................................................ 122
ZmPRMT3    ASVGTRVHGLACWFDVLFNGSTVQRWLTTAPGSPTTHWYQLRCVLSQPLYVMAGQEITGRLHLVAHSAQSYTIYLTMSAK 460
ZmPRMT4    ASVGTRVHGLACWFDVLFNGSTVQRWLTTAPGSPTTHWYQLRCVLSQPLYVMAGQEITGHLRLVAHSAQSYTIYLTMSAK 475
ZmPRMT5    SMMLGELCYSNHNLLPVF.............................................................. 288
ZmPRMT6    SMLQGVT......................................................................... 179
ZmPRMT7    SMLQAPLHGFAFWFDVEFNGPVRQKSKKQPSQSLDENTQNASPSNRKRKPDVSIVLSTAPEDAPTHWQQTLLYLFEPIEL 361
ZmPRMT8    ................................................................................ 144
Consensus

ZmPRMT1    .............................................................................    122
ZmPRMT2    .............................................................................    122
ZmPRMT3    MWGVGAEQGGILQTSTGKLELKEPYYRLSQPQSYMLPQDQQQQQQLSSLQPHGSEQQMQEGLSPGITIDQVDKDYGL    537
ZmPRMT4    MWGVGAEQGGILQTSTGKLELKEPYYRLSQPQSCTLPQDQQQQQ.LPSLQAQGSEQQMQEGLSPAFTIDQDCLN...    548
ZmPRMT5    .............................................................................    288
ZmPRMT6    .............................................................................    179
ZmPRMT7    NKDQIIEGSVTISQSQ.............................................................    377
ZmPRMT8    .............................................................................    144
Consensus
```

Table S1 The information of PRMT genes in plants

Name	Gene ID
ZmPRMT1	Zm00001d015228_T003
ZmPRMT2	Zm00001d054001_T001
ZmPRMT3	Zm00001d022469_T005
ZmPRMT4	Zm00001d007133_T002
ZmPRMT5	Zm00001d026614_T002
ZmPRMT6	Zm00001d032633_T001
ZmPRMT7	Zm00001d036131_T001
ZmPRMT8	Zm00001d020188_T004
AtPRMT1A	AT2G19670.1
AtPRMT3	AT3G12270.1
AtPRMT4A	AT5G49020.2
AtPRMT4B	AT3G06930.2
AtPRMT5	AT4G31120.1
AtPRMT6	AT3G20020.1
AtPRMT10	AT1G04870.2
AtPRMT11	AT4G29510.1
OsPRMT5	LOC_Os02g04660
OsPRMT10	LOC_Os06g05090
SbPRMT5	SORBI_3004G036700
SbPRMT10	SORBI_3010G032700

Table S2 The signal values for maize PRMT genes in the 60 tissues

Gene Name / Tissues	ZmPRMT1	ZmPRMT2	ZmPRMT3	ZmPRMT4	ZmPRMT5	ZmPRMT6	ZmPRMT7	ZmPRMT8
**24H_Germinating Seed**	10.64	11.63	10.52	12.73	11.57	12.1	11.52	12.23
**6DAS_GH_Coleoptile**	12.79	13.32	10.24	12.75	12.32	12.22	12.52	12.73
**6DAS_GH_Primary Root**	12.52	13.25	10.13	12.6	11.78	12	12.29	12.61
**V1_GH_Primary Root**	11.71	12.72	9.89	12.38	10.94	11.56	11.48	11.88
**VE_Whole Seedling**	12.43	11.62	10.32	12.12	11.33	11.89	11.76	11.64
**VE_Primary Root**	12.23	12.25	10.2	13.07	11.83	12.75	12.41	12.85
**V1_Pooled Leaves**	11.65	11.01	10.59	11.38	8.63	11.52	9.96	9.35
**V1_Stem and SAM**	13.07	12.44	9.96	13.17	12.27	12.53	12.96	13.18
**V4_Stem and SAM_R3**	12.32	11.71	10.16	12.41	11.76	12.12	11.52	12.18
**V3_Stem and**	12.84	12.39	10.24	12.99	12.02	12.43	12.57	12.75

**SAM**								
**V3_First Leaf and Sheath**	10.88	11.48	9.95	11.94	10.1	11.82	10.96	11.59
**V3_Topmost Leaf**	11.93	11.42	9.85	11.73	9.59	11.79	10.82	10.97
**V5_Shoot Tip**	12.66	12.32	10.62	12.7	12.24	12.55	12.76	12.57
**V5_First Internode**	12.5	12.1	9.86	12.34	12.01	12.36	12.21	12.05
**V5_Tip of stage-2 Leaf**	11.58	11.43	10.27	11.58	10.67	11.58	10.84	10.84
**V5_Base of stage-2 Leaf**	12.52	11.47	10.6	11.93	11.5	11.78	11.91	11.56
**V7_First Internode**	11.83	11.42	10.21	12.32	11.13	12.1	10.84	11.47
**V7_Tip of stage-2 Leaf**	10.92	10.9	10.81	11.98	7.62	11.6	9.44	10.97
**V7_Base of stage-2 Leaf**	12.81	12.01	10.01	12.64	12.16	12.07	11.91	12.14
**V9_Fourth Internode**	13.07	12.2	10.55	12.46	12.34	12.4	12.36	12.34
**V9_Eighth Leaf**	11.07	11.61	10.58	11.87	9.6	11.49	10.35	10.89
**V9_Eleventh Leaf**	11.9	11.72	10.42	11.92	10.17	11.76	10.75	10.66
**V9_Thirteenth Leaf**	12.2	11.77	10.11	12.09	10.88	11.83	10.71	10.99
**V9_Immature Leaves**	12.47	11.32	10.91	12.09	11.2	11.81	11.35	10.93
**V13_Immature Tassel**	12.82	12.45	11.01	12.91	12.68	12.7	12.37	12.74
**V18_Meiotic Tassel**	11.31	11.17	9.91	11.94	10.87	11.81	10.52	11.26
**V18_Immature Cob**	12.67	12.59	11.14	13.06	13.16	12.49	12.06	12.33
**VT_Thirteenth Leaf**	11.49	11.23	10.64	11.51	10.31	11.76	9.96	9.99
**R1_Pre-pollination Cob**	12.64	12.28	10.6	12.19	12.43	12.33	12.43	11.97
**R1_Silks**	12.52	10.74	11.17	12.04	12.35	12.45	12.18	11.45
**R1_Anthers**	11.25	11.47	10.13	11.4	10.71	12.26	9.89	10.85
**R1_Innermost Husk**	11.82	11.48	9.88	12.23	11.4	11.94	11.37	11.07
**R2_Thirteenth Leaf**	11.67	11.4	10.11	11.88	10.81	12.02	10.17	10.79
**R2_Outer Husk**	11.63	11.88	10.04	12.32	11.49	11.87	11.39	11.35
**R2_Innermost Husk**	11.56	11.89	10.09	12.39	11.51	11.72	11.02	11.38

**2DAP_Whole Seed**	12.42	12.18	10.34	12.23	12.29	12.42	12.27	12.05
**4DAP_Whole Seed**	12.42	12.07	10.12	12.08	12.37	12.2	11.74	11.67
**6DAP_Whole Seed**	12.13	12.02	10.15	12.44	12.34	12.63	11.38	11.72
**8DAP_Whole Seed**	12.37	12.13	10.21	12.46	12.22	12.46	11.84	11.64
**10DAP_Whole Seed**	12.41	11.95	10.76	12.71	12.17	12.57	12.22	12.23
**12DAP_Whole Seed**	13.17	12.07	10.42	12.93	12.45	12.88	12.73	12.77
**12DAP_Endopsperm**	13.57	12.66	10.76	12.8	12.73	13.31	13.38	13.33
**14DAP_Whole Seed**	13.33	12.04	10.37	12.27	12.07	12.71	12.93	12.91
**14DAP_Endosperm**	13.35	12.5	10.51	12.22	12.16	12.94	13.19	13.16
**16DAP_Whole Seed**	13.47	12.34	10.73	12.19	12.33	12.55	12.66	12.5
**16DAP_Endosperm**	13.71	12.65	10.75	12.22	11.91	12.89	12.99	13.18
**16DAP_Embryo**	12.74	12.57	11.01	13.1	13.15	12.66	12.74	12.71
**18DAP_Whole Seed**	13.19	12.19	10.23	11.99	11.54	12.27	12.28	12.55
**18DAP_Endosperm**	13.52	12.27	10.61	12.07	11.61	12.68	12.64	12.92
**18DAP_Embryo**	12.56	12.55	10.83	13.1	13.14	12.85	12.76	12.81
**18DAP_Pericarp**	11.94	11.56	10.64	12.3	12.01	12.33	11.29	11.25
**20DAP_Whole Seed**	13.14	12.36	10.53	12.08	11.68	12.69	12.17	12.53
**20DAP_Endosperm**	13.37	12.52	10.52	12.06	11.08	12.94	12.38	12.79
**20DAP_Embryo**	12.69	12.56	10.73	13.03	12.94	12.59	12.22	12.55
**22DAP_Whole Seed**	13.19	12.33	10.28	12.16	11.61	12.87	11.58	12.17
**22DAP_Endosperm**	13.36	12.47	10.51	12.4	10.94	13.03	11.88	12.68
**22DAP_Embryo**	12.99	12.76	11.15	12.75	13.1	12.83	11.86	12.12
**24DAP_Whole Seed**	13	12.25	10.14	12.21	11.61	12.65	11.78	12.22
**24DAP_Endosperm**	13.12	12.14	10.31	12.09	10.27	12.58	11.79	12.29
**24DAP_Embryo**	13.02	12.69	10.48	12.69	12.94	12.75	11.5	12.34

Table S3 Primer sequences for qRT-PCR

Gene name	Forward primer sequence(5’ →3’)	Reverse primer sequence(5’ →3’)
ZmPRMT1	AATCTCTGGGAGATCGAATCAC	ACATCCTTCTCAAAAGTCTCGT
ZmPRMT2	GAAGGGTCTCCTATAGAAGTGC	TTCTCTGTTACAACACAAAGCG
ZmPRMT3	ATTAGTGGAAAAGCTGGTGAGA	TTGCTGATGTAATAATTGCCCG
ZmPRMT4	CCAGAGAAAGCTGACATACTGA	GTATTCGTCAGAAAATGGAGCC
ZmPRMT5	CAAGCGCTTCGATTGCTATAAT	GGATCCGGGAAATACAAAATGG
ZmPRMT6	GCTCATTCATTGTAACGCTTCT	ATGCATTCTACTTGTCCAGACA
ZmPRMT7	CATCCAAACCAAGTTATCGGTC	TTCTGATTGGCAATGTGACTTG
ZmPRMT8	TCCAGTCCCAAAAGTAGATGTC	CAAGCCATTTATCACGTGCATA
ZmActin1	CCTCACCGACCACCTAATG	CCATCAGGCATCTCGTAGC
AtP5CS1	AGAGTCAATGGTGGCTCGCTTAGT	ACACGGCCGATTGGATCTTCCATA
AtP5CR	AAGATTCTGGTTTCTGTTGC	AACGGTCTGTGAAGCTAAA
AtPDH	ATTTGGCAGTACAAAATGCT	AGGACAATGCATCTGACATA
AtP5CDH	CTCAAAGCTACTCTTTGGTG	TCGTAGATGACTTCTCTGTG
AtFLC	CCTAATTTGATCCTCAGGTTTGGG	CCGACGAAGAAAAAGTAGATAGGCAC
AtFT	GAGACCCTCTTATAGTAAGCAGAGTTG	GGGAGTTCAAGTGAAAGAACCAAAGT
AtSOC1	GCTCTCAGTGCTTTGTGATGC	A AGAACGTACTTGGAGCTGGC
AtMAF1	GGAAAGAATACGTTGCTGGCAACA	CCGTTGATGATGGTGGCTAATTGA
AtMAF2	GGCTCCGGAAAACTCTACAA	TTCTGCAAGATCTAAGGCTTCA
AtMAF3	ACAGAACTAATGATGGAGGATATGAA	CTTCTTCCCCACCTGGCT
AtMAF4	GAGCAATGTCACCGGAAAGTAG	CAGTCGTTGGTGATGGTGGTTA
AtMAF5	AAGAGCAGTAATGTCACCGGAA	ACTTGAGAAGCGGGAGAGTC
AtActin1	CTTGCACCAAGCAGCATGAA	CCGATCCAGACACTGTACTTCCTT

Table S4 Primer sequences for clone-PCR

Gene name	Forward primer sequence(5’ →3’)	Reverse primer sequence(5’ →3’)
ZmPRMT1	CCC<u>AAGCTT</u>ATGATTTTGACATTGC	TGC<u>TCTAGA</u>TTATAGACCAACCCAATAG G
